# Saunders’s Question Compends

**Published:** 1901-02

**Authors:** 


SAUNDERS'S QUESTION COMPENDS. Essentials of Histology. By LOUIS LEROY, B. S., M. D., Professor of Histology and Pathology, in Vanderbilt University, Medical and Dental Departments; City Bacteriologist to Nashville, Tenn.; Bacteriologist to the State of Tennessee. Arranged with questions following each chapter. Duodecimo, pp. 231; 72 illustrations. Philadelphia: W. B. Saunders & Co. 1900. [Price, $1.00 net.]

Since the issue of the first volume of the Saunders's Question compends, over 175,000 copies have been sold. This enormous sale is indisputable evidence of the value of these self helps to students and physicians. Twenty-five volumes have been issued and all enjoy the confidence of the profession as the successive editions testify. The present volume is in its infancy and belongs to the more recent additions to this little library. It is a worthy rival for honors and the author has made of it a very useful and instructive guide. The author has collected within a limited space and in convenient form the essential facts of histology, and at the same time has endeavored not to sacrifice clearness and intelligibility to the necessarily great condensation. The facts are well presented, the illustrations are many and well selected and to the busy practitioner it affords a very handy volume to refer to in his spare moments.

The volume is bound to correspond with the other volumes of this series. W. C. K.

